# Non-destructive OAM measurement via light–matter interaction

**DOI:** 10.1038/s41377-022-00749-0

**Published:** 2022-03-10

**Authors:** Gianluca Ruffato

**Affiliations:** 1 Department of Physics and Astronomy ‘G. Galilei’, University of Padova, Padova, Italy; 2 Quantum Technologies Research Center (QTech), University of Padova, Padova, Italy

**Keywords:** Nonlinear optics, Optical techniques

## Abstract

The detection of orbital angular momentum usually relies on optical techniques, which modify the original beam to convert the information carried on its phase into a specific intensity distribution in output. Moreover, the exploitation of high-intensity beams can result destructive for standard optical elements and setups. A recent publication suggests a solution to overcome all those limitations, by probing highly-intense vortex pulses with a structured reference beam in a strong-field photoionization process.

## Abstract (web summary)

A recent publication suggests a new solution for the in situ non-destructive OAM measurement of highly-intense vortex pulses by analyzing the photoelectrons momentum distribution generated in a strong-field ionization process.

## Graphical abstract

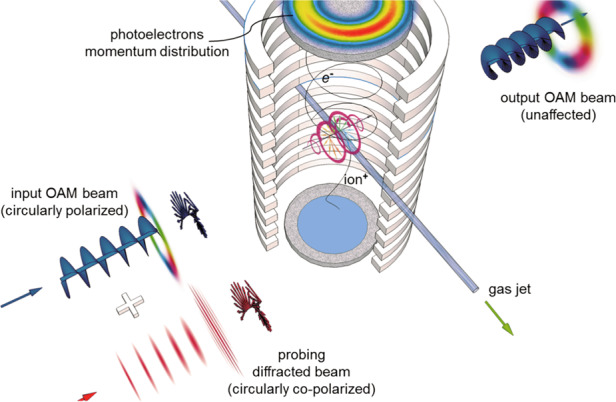

Almost 30 years have passed since the seminal paper of Allen and co-workers^1^ ignited a still vivid field of research, which has paved the way to amazing applications in an unexpected variety of areas, including life science, soft and condensed matter, and information and communication technology. In particular, orbital angular momentum (OAM) beams have provided a new degree of freedom to encode information^2^ or increase the Hilbert space in quantum communications^3^, while their uncommon phase and intensity distributions have enabled advanced techniques in micromanipulation^4^, imaging^5^, and light–matter interaction^6^. Concurrently, those research and applications have inspired the development of new optical components and innovative methods for the controlled generation, manipulation, and measurement of optical angular momentum.

In particular, the measurement of OAM can rely on a rich portfolio of optical elements and architectures with different levels of complexity, integration, and miniaturization, such as interferometric^7^ or diffractive^8^ techniques, holographic methods^9^, conformal mappings^10,11^ multiplane light conversion^12^, and integrated photonics^13^, to name the most used. However, all those solutions inevitably modify the input beam to obtain an output intensity distribution, which is correlated to the input amount of OAM. In addition, the required physical processing of the input wavefront prevents the application of those methods to high-intensity beams, which may damage or even destroy the optical elements.

A recent experiment coordinated by Professor Y. Liu^14^ suggests a possible way-out to detect the OAM of an intense optical vortex, by analyzing the spatial distribution of the electrons emitted in a strong-field photoionization process^15^. In such a way, the information on the input field is transferred to secondary carriers, i.e., photoelectrons, with the sacrifice of a negligible amount of photons from the original beam. Thus, from the analysis of the photoelectron momentum distribution or angle-resolved yields, it is possible to infer the amount of OAM carried by the probed optical vortex without affecting significantly its original phase and intensity distribution.

In this work, a two-color co-rotating circular laser field is synthesized by the combination of circularly polarized probing pulses at 800 nm with co-polarized 400-nm optical vortices produced by the cascade of a frequency-doubling non-linear crystal (*β*-BBO) with a spiral phase plate for OAM generation. The synthesized field is made to interact with a supersonic Argon atom gas jet in a vacuum chamber, and a time-of-flight spectrometer with a position-sensitive detector is used for the three-dimensional reconstruction of photoelectron momentum distribution^16^ (Fig. 1). In particular, by inserting a slit in order to spatially sculpture the probing field via diffraction, the authors show how the consequent spatial structuring of the synthesized field induces an axial asymmetry in the photoelectron distribution, which can be correlated to the OAM amount of the unknown vortex field. Moreover, when the slit is rotated in time, the yields of photoelectrons confined within a defined range exhibits a periodic trend, with the peaks’ number and position depending on the value and sign of the probed OAM.

**Fig. 1:**
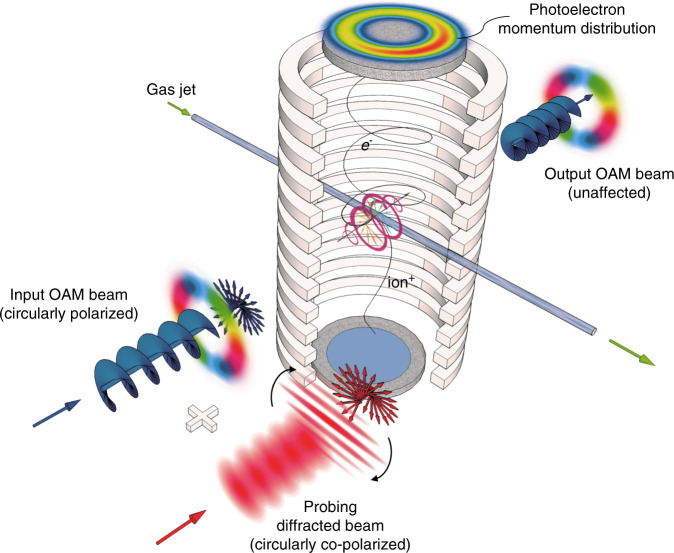
Pictorial scheme of non-destructive OAM measurement set-up via intense-light–matter interaction^14^. Circularly polarized OAM pulses (400-nm, OAM equal to 2) are made to interfere with co-polarized structured pulses (800-nm) obtained by single-slit diffraction. The resulting two-color co-rotating circular field interacts with a supersonic argon atom gas jet inside a vacuum chamber under applied magnetic and electric fields, inducing photoionization. The OAM of the unknown vortex pulses can be inferred from the reconstructed photoelectron momentum distribution

This solution prevents the OAM state from collapsing as in previous methods, so that the measured vortex pulses can be then exploited in subsequent applications, while avoiding at the same time the passage through physical optical elements, which could result in irreparable damage by the intense field.

While the authors provide an experimental proof only for low-order states, a universal scheme to detect any amount of OAM is also suggested using the same set-up. The research opens to new scenarios for the analysis of highly-intense vortex fields, suggesting significant implications to the study of the optical angular momentum of ultrafast and intense optical vortices. The availability of an in situ non-destructive analysis method is expected to enable new applications and further extend the study of intense optical vortices in light–matter interaction and structured light dynamics. Future analyses can also include more complex laser fields, such as vector^17^ or skyrmionic^18^ beams, while possible applications can be envisaged to the generation and analysis of structured matter waves, e.g., electron beams^19^.
